# Better Response and Prognosis of Venetoclax Plus Hypomethylating Agents Over Intensive Chemotherapy in Young Adults With Newly Diagnosed *ASXL1* ‐Mutated Acute Myeloid Leukemia

**DOI:** 10.1002/cam4.71037

**Published:** 2025-07-18

**Authors:** Yiming Cai, Jingwen Rui, Zhengwen Ding, Judan Xie, Zhou Jin, Jinyan Xiao, Yang Xu

**Affiliations:** ^1^ National Clinical Research Center for Hematologic Diseases, Jiangsu Institute of Hematology, Jiangsu Key Laboratory of Hematologic Diseases The First Affiliated Hospital of Soochow University Suzhou P. R. China; ^2^ Institute of Blood and Marrow Transplantation, Collaborative Innovation Center of Hematology Soochow University Suzhou P. R. China

**Keywords:** acute myeloid leukemia, *ASXL1*, hypomethylating agents, venetoclax

## Abstract

**Background:**

*ASXL1* is one of the most frequently mutated genes in acute myeloid leukemia (AML) and retains adverse‐risk status in intensively treated cohorts according to 2022 European Leukemia Net (ELN) risk criteria. The therapeutic and prognostic impacts of hypomethylating agents (HMAs) and venetoclax in young adults with *ASXL1*‐mutated AML is unclear.

**Methods:**

Eighty‐one patients with *ASXL1*‐mutated AML ≤ 60 years old were retrospectively analyzed. The effects of HMAs plus venetoclax on treatment response and its prognostic value were compared with intensive chemotherapy (IC) and HMAs combined with low‐intensity chemotherapy.

**Results:**

Intensive chemotherapy independently predicted a worse treatment response (IC vs. HMA + venetoclax, OR = 0.183, 95% CI 0.048–0.693, *p* = 0.012) and inferior overall survival (OS) (IC vs. HMA + venetoclax, HR = 3.316, 95% CI 1.332–8.255, *p* = 0.010). After 15 patients with favorable cytogenetics or mutations were excluded, the HMA + venetoclax combination still outweighed IC with respect to treatment response (IC vs. HMA + venetoclax, OR = 0.063, 95% CI 0.012–0.332, *p* = 0.001) and OS (IC vs. HMA + venetoclax, HR = 3.072, 95% CI 1.216–7.758 *p* = 0.018) in patients with an adverse risk according to 2022 European Leukemia Net guidelines. Allogeneic hematopoietic stem cell transplantation independently predicted superior OS (HR = 0.234, 95% CI 0.088–0.626, *p* = 0.004). Additionally, in patients receiving HMAs combined with venetoclax, the G646fs variant of the *ASXL1* mutation was associated with a lower complete remission or with an incomplete hematological recovery rate (4/7 vs. 2/19, 42.9% vs. 10.5%, *p* = 0.026) and worse event‐free survival (median, 14.0 months vs. not reach, *p* = 0.045).

**Conclusion:**

HMAs and venetoclax could benefit newly diagnosed younger patients with *ASXL1*‐mutated AML.

## 1 Introduction

Acute myeloid leukemia (AML) is a heterogeneous disease characterized by diverse driver mutations and clone evolution. The additional sex comb‐like 1 gene (*ASXL1*), which occurs in approximately 3%–10% of patients [1, 2], is one of the most frequently mutated genes in AML. It encodes a chromatin‐binding protein of the polycomb group and trithorax complex family, and mutated *ASXL1* leads to extensive epigenetic dysregulation. While the underlying mechanism of leukemogenesis caused by *ASXL1* mutation has not been fully elucidated, its prognostic value for AML has been reported. According to the 2017 and 2022 European Leukemia Net (ELN) risk stratifications, mutated *ASXL1* defines an adverse‐risk subgroup of AML except for the coexistence with favorable‐risk AML subtypes [3, 4].

Traditional “3 + 7” intensive chemotherapy (IC) remains the cornerstone of treatment for AML. However, for 2022 ELN adverse‐risk AML patients, only a rather unsatisfactory complete remission (CR) rate of 43% to 45% has been reported [5, 6]. In the ALFA‐1200 study in which patients were treated with standard chemotherapy, those who carried secondary AML‐like mutations, including those in *ASXL1*, were associated with a worse prognosis [7]. Notably, the prognostic stratification of ELN is strictly contingent on therapeutic context and the unfavorable prognostic association of *ASXL1* mutations and AML is primarily established in patients undergoing IC. The prognostic value of *ASXL1* mutations is not validated in cohorts receiving nonintensive therapeutic approaches under the genetic stratification of 2024 ELN recommendations [8]. Venetoclax (VEN), a promising BCL‐2 inhibitor, in combination with hypomethylating agents (HMAs) has been shown to be effective in elderly patients who are unfit for IC across all genomic risk groups [9, 10]. However, how well the venetoclax performs in patients ≤ 60 years old with 2022 ELN adverse cytogenetics and high‐risk mutations, including those in *ASXL1*, is not fully understood. In a phase 2 study, venetoclax plus decitabine induction was effective and well tolerated in younger adults with adverse‐risk AML [11]. In addition, Gangat et al. [12] reported that a favorable response was predicted by *ASXL1* mutation among patients treated with first‐line HMAs and venetoclax.

Thus, we studied the prognostic factors of AML patients with *ASXL1* mutations and retrospectively compared the effects of HMAs and venetoclax on patient response and prognosis with those of IC and reduced‐intensity chemotherapy to explore the therapeutic and prognostic impacts of HMA and venetoclax.

## 2 Patients and Methods

### 2.1 Patients

Eighty‐one newly diagnosed AML patients (excluding APL patients) with *ASXL1* mutation (*ASXL1* ^mut^) were retrospectively analyzed. Adult patients under or equal to the age of 60, with a diagnosis made between September 2016 and August 2023, and with available information from next‐generation sequencing at diagnosis were included. Patients with insufficient bone marrow data at diagnosis, lacking post‐induction therapeutic response assessments, or with a diagnosis of concurrent malignancies were excluded. The study protocol was performed in accordance with the Declaration of Helsinki, and an exemption was granted by the Ethics Committee of the First Affiliated Hospital of Soochow University.

### 2.2 Treatments

All patients received treatment according to the Chinese guidelines for the diagnosis and treatment of adult acute myeloid leukemia. For induction, each of 30 patients received a hypomethylating agent (HMA) and venetoclax (azacitidine 75 mg/m2 d1‐7 or decitabine 20 mg/m^2^ d1‐5, venetoclax dose ramping up: 100 mg d1, 200 mg d2, 400 mg d3‐28). Thirty‐two fit patients received intensive chemotherapy (idarubicin, 8–12 mg/m^2^ d1‐3; and cytarabine, 100 mg/m^2^ d1‐7). Nineteen patients received HMAs combined with low‐intensity chemotherapy primed with granulocyte colony stimulating factor (G‐CSF) (HMA + IAG, azacitidine 75 mg/m^2^ d1‐7 or decitabine 20 mg/m^2^ d1‐5, idarubicin 5 mg/d d4‐8, cytarabine 20 mg/m^2^ d4‐10, and G‐CSF adjusted to count blood cells; HMA + HAG, azacitidine 75 mg/m^2^ d1‐7 or decitabine 20 mg/m^2^ d1‐5, homoharringtonine, 2 mg/d d1‐7, cytarabine 20 mg/m^2^ d1‐7, and G‐CSF adjusted to count blood cells). All patients achieved CR/CRi following IC or HMA + IAG/HAG induction underwent consolidation therapy with either intermediate to high dose cytarabine (1–3 g/m^2^ d1‐3) or cytarabine (1–2 g/m^2^ d1‐3)‐based combination chemotherapy (HMA + IAG/HAG). CR/CRi patients initiated with HMA and venetoclax continued the original regimen or underwent consolidation therapy of cytarabine‐based regimen according to patients' comorbidities and performance status. Allogeneic hematopoietic stem cell transplantation (allo‐HSCT) was based on disease status and potential donors. Patients who did not achieve CR or CRi after the first induction cycle continued the initial regimen or transitioned to other regimens (IC, HMA + venetoclax or HMA + HAG/IAG) aligned with Chinese guidelines and individualized clinical assessments. Salvage transplantation was performed according to disease status and donor availability.

### 2.3 Next‐Generation Sequencing

Genomic DNA was extracted from the bone marrow of patients at the time of diagnosis. The Ion S5 Personal Genome Machine (Thermo Fisher Scientific, Waltham, MA) was used to evaluate a panel of 51 or 172 genes recurrently mutated in myeloid malignancies. The variant allele fraction (VAF) was the proportion of variant alleles within a genomic locus. If two or more ASXL1 mutations were present in the same patient, the larger VAF was used for analysis.

### 2.4 Definitions of Response and Outcome Measures

CR was defined as < 5% bone marrow blasts, no blasts in the blood, absolute neutrophil counts ≥ 1.0 × 10^9^/L, platelet counts ≥ 100 × 10^9^/L, and no extramedullary disease. CR with incomplete hematological recovery (CRi) was defined as all CR criteria met except for residual neutropenia < 10^9^/L or thrombocytopenia < 100 × 10^9^/L. Partial remission (PR) was defined as all hematologic criteria of CR, a decrease of bone marrow blast percentage to 5%–25%, and a decrease of pretreatment bone marrow blast percentage by at least 50%. Overall remission (OR) was defined as all criteria of CR/CRi or PR. Relapse was measured as the reappearance of blasts in the blood or bone marrow (≥ 5%) or an extramedullary site after CR. Overall survival (OS) was calculated from diagnosis to death or last follow‐up. Relapse‐free survival (RFS) was calculated from the first CR to relapse, with censoring at death in CR, or the last follow‐up. Event‐free survival (EFS) was measured from diagnosis to treatment failure, relapse, or death. Patients were censored at allo‐HSCT when necessary. The cutoff point of variant allele frequency (VAF) was defined by the median (32.70%) of all patients, and they were categorized into ASXL1‐low or ASXL1‐high group.

### 2.5 Statistical Analysis

Categorical variables were analyzed by the chi‐squared test or Fisher's exact test. Continuous variables were analyzed by the Mann–Whitney U test. Risk factors for response were calculated via logistic regression. OS, EFS, and RFS were estimated via the Kaplan–Meier method, and differences were calculated via the log‐rank test. Survival curves of transplantation were conducted by landmark analysis with a landmark time of 6 months. Survival models were performed via Cox regression. The covariates with a *p* value < 0.15 in the univariate analysis were included in the multivariate model for prognosis. Allo‐HSCT was considered a time‐dependent covariate in the survival model. Two‐sided *p* < 0.05 was considered statistically significant. Statistical analyses were conducted using SPSS (version 26.0; IBM Corp.) and R (version 4.4.2; R Foundation for Statistical Computing), while figures were generated with R 4.4.2 and GraphPad Prism (version 10.0.2; GraphPad Software).

## 3 Results

### 3.1 Clinical and Genetic Characteristics of ASXL1^mut^ AML

From September 2016 to August 2023, 81 patients aged between 18 and 60 years with *ASXL1* ^mut^ AML were included. Forty‐nine (60.5%) patients were male, and 32 (39.5%) were female. The median age at diagnosis was 50 years. As shown in Table 1, patients were classified into three groups by their induction therapy, with 30 receiving HMA–venetoclax, 32 receiving intensive chemotherapy, and 19 receiving HMA plus IAG or HAG. Sixty‐six (81.5%) patients were at adverse risk according to the 2022 ELN guidelines, and because harboring favorable cytogenetics or mutations, 15 (18.5%) patients were considered at nonadverse risk. Twelve (12/81, 14.8%) patients presented with *t* (8;21) or *RUNX1::RUNX1T1*, a favorable‐risk fusion gene reported to be strongly associated with ASXL1 mutation [13]. This frequency of *RUNX1::RUNX1T1* was similar to that reported in another retrospective study of 91 patients with ASXL1^mut^ AML [14]. Owing to the reportedly limited effectiveness of HMA plus venetoclax in patients harboring *RUNX1::RUNX1T1* [15], only one patient received HMA–venetoclax, and 8 received IC in this study, which led to a statistically significant difference in ELN risk classification among these three treatment groups (2022 ELN adverse risk, HMA + VEN vs. IC vs. HMA + IAG/HAG, 96.7% vs. 64.5% vs. 84.2%, *p* = 0.003). No significant differences in age, sex, blast count, VAF of *ASXL1* at diagnosis, other cytogenetic aberrations, and number of patients undergoing allo‐HSCT were found among the three groups (Table 1).

**TABLE 1:** Comparison of clinical characteristics of 81 patients with *ASXL1*‐mutated AML grouped by induction regimen.

Group	All *n* = 81	HMA + VEN *n* = 30	IC *n* = 32	HMA + IAG/HAG *n* = 19	*p*
Age (y)					
Median (range)	50 (18–60)	47 (19–60)	50 (18–60)	53 (32–60)	0.067
Gender					
Male	49 (60.5%)	19 (63.3%)	19 (59.4%)	11 (57.9%)	0.918
Female	32 (39.5%)	11 (36.7%)	13 (40.6%)	8 (42.1%)	
AML type					
De novo AML	73 (90.1%)	27 (90%)	32 (100%)	14 (73.7%)	0.050
Secondary AML	8 (9.9%)	3 (10%)	0	5 (26.3%)	
WBC (10^9^/L)					
Median (range)	11.00 (0.66–190.00)	12.76 (0.67–190.00)	10.46 (0.66–111.32)	6.52 (1.10–116.3)	0.396
PLT (10^9^/L)					
Median (range)	51 (4–552)	72.5 (12–552)	38 (6–164)	59 (4–198)	**0.023**
Hb (g/L)					
Median (range)	57 (32–149)	82 (41–149)	71 (32–149)	79 (44–142)	0.324
BM blast (%)					
Median (range)	50 (11.5–97.0)	44.0 (12.0–96.2)	49.0 (13.0–97.0)	50 (12–97)	0.773
Cytogenetic abnormalities					
*t* (8;21) or *RUNX1::RUNX1T1*	12 (14.8%)	1 (3.3%)	8 (25.8%)	3 (15.8%)	**0.049**
MDS‐related cytogenetic abnormalities	18 (22.5%)	5 (16.7%)	8 (25.8%)	5 (26.3%)	0.625
Adverse cytogenetics	11 (13.9%)	5 (16.7%)	3 (9.7%)	3 (15.8%)	0.681
2022 ELN					
Nonadverse risk	15 (18.5%)	1 (3.3%)	11 (35.5%)	3 (15.8%)	**0.003**
Adverse risk	66 (81.5%)	29 (96.7%)	20 (64.5%)	16 (84.2%)	
ASXL1 VAF (%)					
Median (range)	32.7 (1.26–53.5)	33.6 (4.0–52.6)	37.4 (1.4–53.5)	26.56 (1.26–45.31)	0.153
Hotspot of ASXL1 mutation					0.927
G646fs	16 (19.8%)	6 (20.0%)	6 (18.7%)	4 (21.1%)	
E635fs	10 (12.3%)	5 (16.7%)	3 (9.4%)	2 (10.5%)	
Other spots	42 (51.9%)	15 (50.0%)	16 (50.0%)	11 (57.9%)	
Unavailable	13 (16.0%)	4 (13.3%)	7 (21.9%)	2 (10.5%)	
allo‐HSCT					
Number (%)	41 (50.6%)	20 (66.7%)	14 (43.7%)	7 (36.8%)	0.077

*Note:* Bold values indicate statistically significant differences (*p* < 0.05).

Abbreviations: allo‐HSCT, allogeneic hematopoietic stem cell transplantation; AML, acute myeloid leukemia; ELN, European Leukemia Net; HAG, homoharringtonine and cytarabine, primed with granulocyte colony stimulating factor; HMA, hypomethylating agent; IAG, idarubicin and cytarabine, primed with granulocyte colony stimulating factor; IC, intensive chemotherapy; MDS, myelodysplastic syndromes; VEN, venetoclax.

Sixty‐eight patients had available details of *ASXL1* mutations. One patient harbored two mutated sites in *ASXL1*. The median VAF of mutated *ASXL1* was 32.70% (range, 1.3%–51.0%). Found in 16 (16/68, 23.5%) patients, G646fs was the most common mutated hotspot (Table 1). Additional mutations were identified in 73 (90.1%) of the 81 *ASXL1* ^mut^ patients (Figure S1a). The most prevalent comutated genes were *RUNX1* (21/73, 28.8%), *IDH2* (19/73, 26.0%), *U2AF1* (18/73, 24.6%), *NRAS* (16/73, 16.1%), and *TET2* (12/73, 16.4%) (Figure S1c). Mutations involved in transcription factors (35/81, 43.2%) and activated signaling (33/81, 40.7%) were mostly prevalent (Figure S1b).

### 3.2 Response to Induction in ASXL1^mut^ AML

Eighty‐one patients were able to be evaluated for their response to induction therapy after the first cycle. OR was achieved in 59 patients (59/81, 72.8%), 44 of whom achieved CR/CRi (44/81, 54.3%). Among those receiving HMAs combined with venetoclax, 23 (76.7%) patients achieved CR/CRi, which was significantly higher than that reported in the other two groups (23/30 vs. 14/32 vs. 7/19, 76.7% vs. 43.8% vs. 36.8%, *p* = 0.01). Among the patients with CR/CRi and available data on measurable residual disease (MRD), 24 (24/41, 61.5%) patients had an MRD of less than 10^−3^ by multiparameter flow cytometry after the first cycle of treatment, 14 (14/21, 66.7%) of whom received HMA–venetoclax induction, whereas 6 (6/11, 54.5%) and 4 (4/7, 57.1%) patients were treated with IC and HMA plus HAG or IAG, respectively (*p* = 0.747).

Thirty‐seven patients did not achieve CR/CRi in the first cycle. A total of six patients did not continue treatment in our center due to physical condition and patients' preference. Among the remaining 31 evaluable patients, 12 received HMA–venetoclax as second‐line therapy, with CR/CRi in seven cases (58.3%). Ten patients received IC‐based salvage regimens, with CR/CRi achieved in only one case (10.0%). Nine patients were treated with HMA + IAG/HAG, resulting in CR/CRi in three cases (33.3%). Overall, 11 out of 31 patients (35.5%) achieved CR/CRi following the second cycle of treatment. Notably, among the 14 patients who failed to achieve CR/CRi after the first IC cycle, four received HMA–venetoclax as second‐line therapy, and all of them achieved CR/CRi.

### 3.3 Therapeutic Impact of HMA‐Venetoclax on ASXL1^mut^ AML

To identify independent risk factors and the therapeutic impact of HMAs and venetoclax, a response analysis for CR/CRi after the first cycle was performed via logistic regression. In the univariate analysis, compared with HMA–venetoclax induction, the IC (OR = 0.237, 95% CI 0.079–0.709, *p* = 0.010) and HMA + IAG/HAG regimens (OR = 0.178, 95% CI 0.050–0.625, *p* = 0.007) were less likely to lead to a CR/CRi. Female sex (OR = 3.407, 95% CI 1.310–8.864, *p* = 0.012) was associated with a better CR/CRi rate. A multivariate analysis was then performed to determine whether HMA plus venetoclax was an independent factor for a better response. Variables with *p* < 0.15 were included in the multivariate model calculated via logistic regression, including sex, 2022 ELN, induction therapy, mutation type of *ASXL1*, mutation of *RUNX1*, *N/KRAS*, *U2AF1*, and *ETV6*. Compared with HMAs combined with venetoclax, both the IC (OR = 0.183, 95% CI 0.048–0.693, *p* = 0.012) and HMA + IAG/HAG (OR = 0.202, 95% CI 0.050–0.819, *p* = 0.025) independently predicted a worse response in ASXL1^mut^ AML patients. We also found that sex was a risk factor for CR1, as female patients had a better response than male patients did (OR = 4.273, 95% CI 1.300–14.041, *p* = 0.017) (Table 2).

**TABLE 2:** Univariate and multivariate analysis for CR/CRi after the first cycle in patients with *ASXL1*‐mutated AML.

Variate	Univariate		Multivariate	
	OR (95% CI)	*p*	OR (95% CI)	*p*
Sex				
Female vs. Male	3.407 (1.310–8.864)	**0.012**	4.273 (1.300–14.041)	**0.017**
2022 ELN				
Adverse vs. Nonadverse	0.364 (0.105–1.259)	0.110	0.643 (0.100–4.139)	0.642
Induction regimen		**0.010**		**0.022**
IC vs. HMA + VEN	0.237 (0.079–0.709)	**0.010**	0.183 (0.048–0.693)	**0.012**
HMA + IAG/HAG vs. HMA + VEN	0.178 (0.050–0.625)	**0.007**	0.202 (0.050–0.819)	**0.025**
*ASXL1* mutation type				
Nonsense vs. Frameshift	0.402 (0.118–1.375)	0.146	0.377 (0.087–1.637)	0.193
*N/KRA*S mutation				
Yes vs. No	0.430 (0.147–1.260)	0.124	0.291 (0.078–1.085)	0.066
*RUNX1* mutation				
Yes vs. No	0.311 (0.109–0.885)	**0.029**	0.398 (0.103–1.534)	0.181
*U2AF1* mutation				
Yes vs. No	0.430 (0.147–1.260)	0.124	0.430 (0.105–1.762)	0.241
*ETV6* mutation				
Yes vs. No	0.204 (0.040–1.052)	0.057	0.745 (0.055–10.045)	0.825

*Note:* Bold values indicate statistically significant differences (*p* < 0.05).

Abbreviations: AML, acute myeloid leukemia; CI, Confidence interval; CR, complete remission; CRi, complete remission with incomplete hematological recovery; ELN, European Leukemia Net; HAG, homoharringtonine and cytarabine, primed with granulocyte colony stimulating factor; HMA, hypomethylating agent; IAG, idarubicin and cytarabine, primed with granulocyte colony stimulating factor; IC, intensive chemotherapy; OR, odd ratio; VEN, venetoclax.

### 3.4 Clinical Outcomes and Prognostic Factors in ASXL1^mut^ AML Patients

**FIGURE 1:**
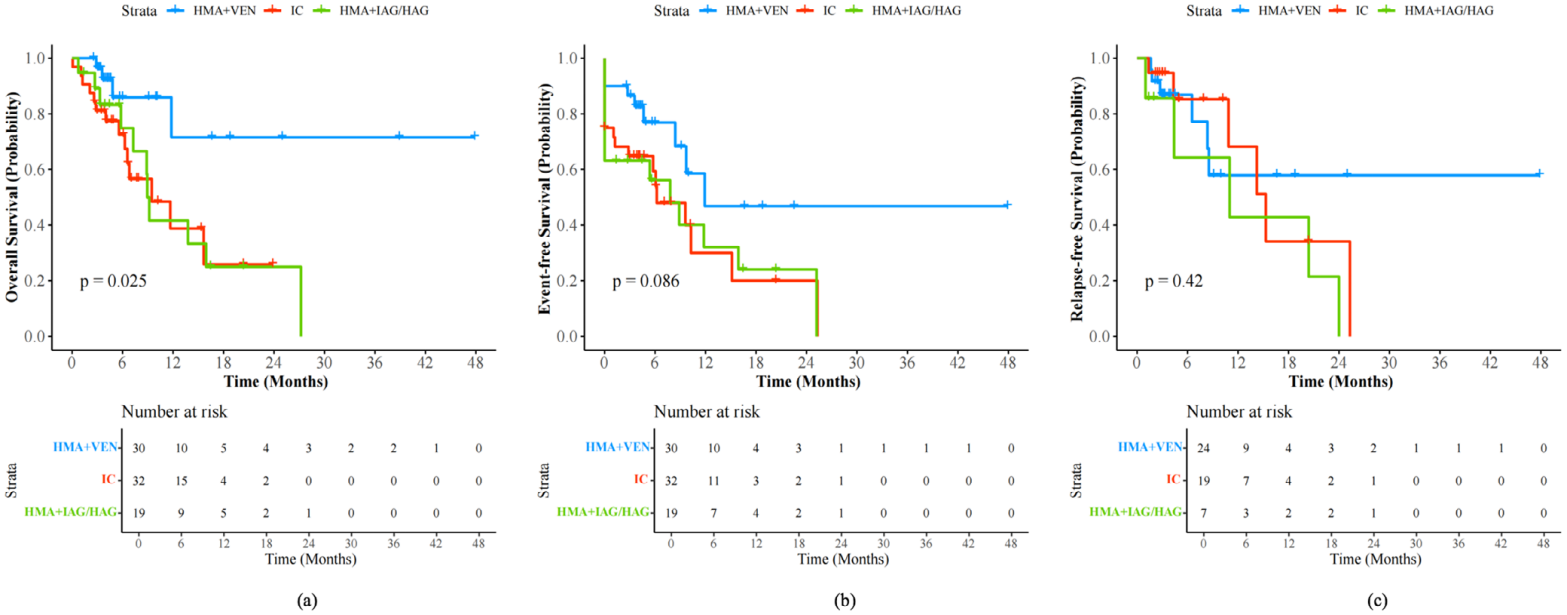
Survival curves of OS (a), EFS (b) and RFS (c) censored at allo‐HSCT in 81 patients with *ASXL1* ^mut^ AML and grouped by the induction therapy. OS, overall survival; EFS, event‐free survival; RFS, relapse‐free survival; allo‐HSCT, allogeneic hematopoietic stem cell transplantation; *ASXL1* ^mut^, *ASXL1* mutation; AML, acute myeloid leukemia. IC, intensive chemotherapy; HMA, hypomethylating agents; VEN, venetoclax; IAG, idarubicin, cytarabine, and granulocyte colony stimulating factor; HAG, homoharringtonine, cytarabine, and granulocyte colony stimulating factor.

The median follow‐up time for all 81 patients was 12.0 (0.7–92.6) months, and the median overall survival time was 27.2 months. Compared with those treated with IC or reduced‐intensity chemotherapy, patients who received HMA–venetoclax induction had significantly longer OS (OS, HMA + VEN vs. IC vs. HMA + IAG/HAG, median: NR vs. 11.7 months vs. 8.9 months, *p* = 0.025) and a tendency toward better EFS (EFS, HMA + VEN vs. IC vs. HMA + IAG/HAG, median: 11.9 months vs. 6.2 months vs. 7.8 months, *p* = 0.086). However, the RFS rates among the treatment groups were not significantly different (RFS, HMA + VEN vs. IC vs. HMA + IAG/HAG, median: NR months vs. 15.3 months vs. 11.0 months, *p* = 0.42) (Figure 1). All patients achieving CR/CRi following IC induction (*n* = 14) or HMA + IAG/HAG (*n* = 7) underwent consolidation therapy with cytarabine‐based regimens. Ten patients receiving frontline HMA–venetoclax induction maintained HMA–venetoclax as consolidation and others switched to cytarabine‐based regimens. Compared with patients receiving the cytarabine‐based regimen, patients receiving consolidation of HMA combined with venetoclax did not have a survival advantage in terms of OS (cytarabine‐based regimen vs. HMA + VEN, median, 25.3 months vs. 27.2 months, *p* = 0.357) or RFS (cytarabine‐based regimen vs. HMA + VEN, median, 25.3 months vs. 14.2 months, *p* = 0.754).

**FIGURE 2:**
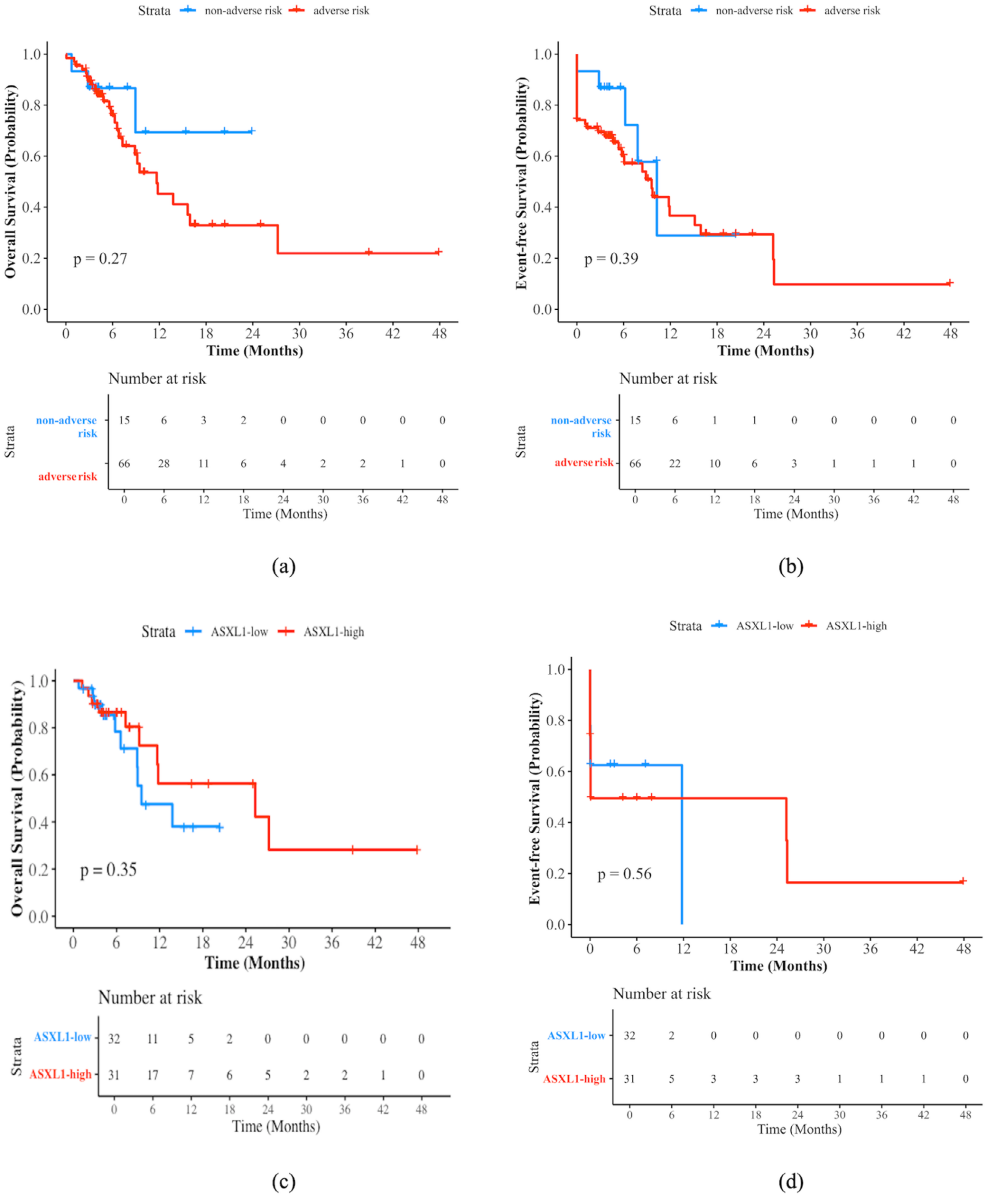
Survival curves of OS (a) and EFS (b) censored at allo‐HSCT in 81 patients with *ASXL1* ^mut^ AML according to 2022 ELN, and survival curves of OS (c) and EFS (d) censored at allo‐HSCT in patients grouped by *ASXL1* VAF. OS, overall survival; EFS, event‐free survival; allo‐HSCT, allogeneic hematopoietic stem cell transplantation; *ASXL1* ^mut^, *ASXL1* mutation; AML, acute myeloid leukemia; ELN, European Leukemia Net.

There were no significant differences in OS or EFS between *ASXL1* ^mut^ patients at nonadverse risk according to the 2022 ELN and those at adverse risk (OS, median, NR vs. 11.8 months, *p* = 0.27; EFS, median, 10.3 months vs. 9.6 months, *p* = 0.39) (Figure 2a,b). Patients were then divided into ASXL1‐high (VAF ≥ 32.7%) and ASXL1‐low (VAF < 32.7%) groups to analyze the impact of the *ASXL1* load on prognosis. The two groups had similar OS and EFS (OS, median, 9.5 months vs. 25.3 months, *p* = 0.35; EFS, median, 9.6 months vs. 9.7 months, *p* = 0.56) (Figure 2c,d).

To determine the prognostic factors of *ASXL1* ^mut^ AML patients, univariate and multivariate Cox regression analyses of OS and RFS were performed. Relevant factors with a *p* value < 0.15 in the univariate analysis were included in the multivariate model. Compared with IC, HMA–venetoclax induction predicted better OS (IC vs. HMA + VEN, HR = 3.316, 95% CI 1.332–8.255, *p* = 0.010), whereas different consolidation therapies did not significantly affect OS (cytarabine‐based regimen vs. HMA + VEN, HR = 0.807, 95% CI 0.635–2.765, *p* = 0.733) (Table 3). Platelet count was also not associated with OS (HR = 0.997, 95% CI 0.991–1.003, *p* = 0.275). Recently, no independent prognostic factors for RFS in patients with ASXL1 mutations were identified.

**TABLE 3:** COX model for overall survival in 81 patients with *ASXL1*‐mutated AML.

Variate	Univariate		Multivariate	
	HR (95% CI)	*p*	HR (95% CI)	*p*
Age				
≥ 45 y vs. < 45 y	2.972 (1.090–8.108)	**0.033**	1.662 (0.695–3.976)	0.254
2022 ELN				
Adverse vs. Nonadverse	2.396 (0.733–7.827)	0.148	3.430 (1.011–11.639)	**0.048**
Induction regimen		0.054		**0.031**
IC vs. HMA + VEN	2.571 (1.057–6.253)	**0.021**	3.316 (1.332–8.255)	**0.010**
HMA + IAG/HAG vs. HMA + VEN	2.992 (1.176–7.609)	**0.037**	2.731 (1.071–6.964)	**0.035**
Consolidation regimen				
Ara‐c‐based regimen vs. HMA + VEN	0.807 (0.635–2.765)	0.733	/	/
allo‐HSCT				
Yes vs. No	0.270 (0.092–0.790)	**0.001**	0.234 (0.088–0.626)	**0.004**

*Note:* Bold values indicate statistically significant differences (*p* < 0.05).

Abbreviations: allo‐HSCT, allogeneic hematopoietic stem cell transplantation; AML, acute myeloid leukemia; Ara‐C, cytarabine; CI, confidence interval; ELN, European Leukemia Net; HAG, homoharringtonine and cytarabine, primed with granulocyte colony‐stimulating factor; HMA, hypomethylating agent; HR, hazard ratio; IAG, idarubicin and cytarabine, primed with granulocyte colony‐stimulating factor; IC, intensive chemotherapy; VEN, venetoclax.

### 3.5 HMA‐Venetoclax in ASXL1^mut^ AML Patients With an Adverse Risk of ELN

Fifteen patients harboring favorable cytogenetic or genetic mutations (*RUNX1::RUNX1T1* or *t* (8;21), *n* = 12; *CBFβ::MYH11*, *n* = 1; bZIP *CEBPA* mutation, *n* = 2) were excluded. Sixty‐six patients were then categorized as having adverse risk according to the 2022 ELN. CR/CRi after the first cycle was achieved in 23 (23/29, 79.3%), four (4/21, 19.0%), and six (6/16, 37.5%) patients receiving HMA–venetoclax, IC, and HMA plus IAG/HAG, respectively (*p* < 0.001). According to the multivariate model for CR/CRi after the first cycle, IC (OR = 0.063, 95% CI 0.012–0.332, *p* = 0.001) and HMA plus IAG/HAG (OR = 0.196, 95% CI 0.044–0.876, *p* = 0.033) led to significantly lower CR/CRi rates than did HMA–venetoclax. Additionally, female patients had a tendency toward a higher CR/CRi rate (OR = 4.245, 95% CI 1.000–18.028; *p* = 0.050) (Table 4).

**TABLE 4:** Logistic regression for CR/CRi after the first cycle of treatment in 66 patients with adverse‐risk AML subtype.

Variate	Univariate		Multivariate	
	OR (95% CI)	*p*	OR (95% CI)	*p*
Sex				
Female vs. Male	3.095 (1.051–9.113)	**0.040**	4.245 (1.000–18.028)	0.050
Induction regimen		**< 0.001**		**0.003**
IC vs. HMA + VEN	0.061 (0.015–0.252)	**< 0.001**	0.063 (0.012–0.332)	**0.001**
HMA + IAG/HAG vs. HMA + VEN	0.157 (0.040–0.606)	**0.007**	0.196 (0.044–0.876)	**0.033**
*ASXL1* mutation type				
Nonsense vs. Frameshift	0.333 (0.088–1.256)	0.105	0.241 (0.047–1.235)	0.088
Chromatin modifiers				
Mutation vs. Wild‐type	0.398 (0.043–1.112)	0.079	0.352 (0.082–1.508)	0.160
RUNX1				
Mutation vs. Wild‐type	0.414 (0.140–1.230)	0.112	0.249 (0.054–1.160)	0.077

*Note:* Bold values indicate statistically significant differences (*p* < 0.05).

Abbreviations: AML, acute myeloid leukemia; CI, confidence interval; CR, complete remission; CRi, complete remission with incomplete hematological recovery; HAG, homoharringtonine and cytarabine, primed with granulocyte colony stimulating factor; HMA, hypomethylating agent; IAG, idarubicin and cytarabine, primed with granulocyte colony stimulating factor; IC, intensive chemotherapy; OR, odd ratio; VEN, venetoclax.

In the survival model, *ASXL1* ^mut^ patients treated with IC induction had worse OS than HMA–venetoclax patients did (HR = 3.072, 95% CI 1.216–7.758, *p* = 0.018) (Table 5). In addition, complex karyotype was the only prognostic factor associated with adverse RFS (HR = 6.634, 95% CI 1.310–33.583; *p* = 0.022). Neither OS (HR = 1.544, 95% CI 0.398–5.996, *p* = 0.530) nor RFS (HR = 1.220, 95% CI 0.311–4.783, *p* = 0.776) differed according to cytarabine‐based consolidation compared to the combination of venetoclax and HMAs.

**TABLE 5:** Cox model for overall survival in 66 patients with adverse‐risk AML subtype.

Variate	Univariate		Multivariate	
	HR (95% CI)	*p*	HR (95% CI)	*p*
Age				
≥ 45 y vs. < 45 y	3.149 (1.291–7.682)	**0.012**	1.676 (0.624–4.503)	0.305
Hotspot of *ASXL1* mutation				
Other spots vs. G646fs	0.531 (0.240–1.176)	0.119	0.797 (0.337–1.881)	0.604
Induction regimen		**0.019**		0.059
IC vs. HMA + VEN	3.614 (1.472–8.865)	**0.005**	3.072 (1.216–7.758)	**0.018**
HMA + IAG/HAG vs. HMA + VEN	2.713 (1.013–7.138)	**0.043**	1.960 (0.709–5.415)	0.194
Consolidation regimen				
Ara‐c‐based regimen vs. HMA + VEN	1.544 (0.398–5.996)	0.530	/	/
allo‐HSCT				
Yes vs. No	0.244 (0.093–0.635)	**0.004**	0.266 (0.097–0.729)	**0.010**

*Note:* Bold values indicate statistically significant differences (*p* < 0.05).

Abbreviations: allo‐HSCT, allogeneic hematopoietic stem cell transplantation; AML, acute myeloid leukemia; Ara‐C, cytarabine; CI, confidence interval; HAG, homoharringtonine and cytarabine, primed with granulocyte colony‐stimulating factor; HMA, hypomethylating agent; HR, hazard ratio; IAG, idarubicin and cytarabine, primed with granulocyte colony‐stimulating factor; IC, intensive chemotherapy; VEN, venetoclax.

### 3.6 Mutation of G646fs in Patients Receiving HMA‐Venetoclax

**FIGURE 3:**
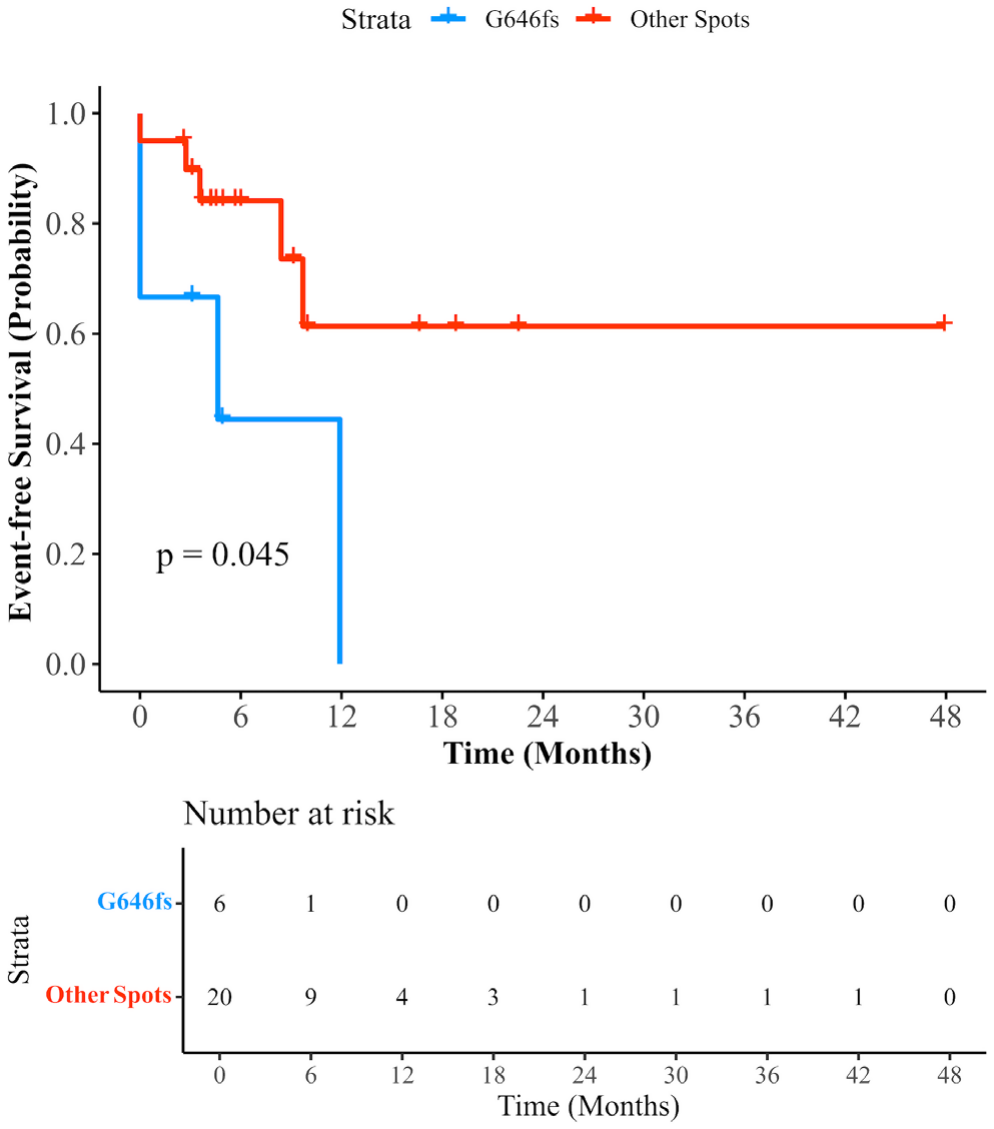
Survival curves of EFS censored at allo‐HSCT in 26 patients receiving HMA‐VEN and grouped by hotspot of *ASXL1* mutations. EFS, event‐free survival; allo‐HSCT, allogeneic hematopoietic stem cell transplantation; HMA, a hypomethylating agent; VEN, venetoclax.

Among the 30 patients who received HMA–venetoclax induction, seven achieved only a PR or NR after the first cycle. Eleven (36.7%) patients continued HMA–venetoclax consolidation, and 20 (66.7%) eventually underwent allo‐HSCT. Twenty‐six patients had available information on hotspots of *ASXL1* mutation. *ASXL1* G646fs was more frequently mutated in patients who did not achieve CR/CRi after the first cycle of HMA–venetoclax induction (4/7 vs. 2/19, 42.9% vs. 10.5%, *p* = 0.026). Two of the 30 patients harbored *SETBP1* mutations, and neither achieved CR/CRi under the induction of HMAs plus venetoclax (*p* = 0.048). No significant differences in age, sex, counts of blood cells and blasts, VAF of *ASXL1* at diagnosis, or cytogenetic or other genetic aberrations were found between CR/CRi and non‐CR/CRi patients. In addition, patients with mutated G646fs had significantly shorter EFS than those with other mutated hotspots (G646fs vs. other hotspots, median, 4.6 months vs. NR, *p* = 0.045) (Figure 3).

### 3.7 Allo‐HSCT in *ASXL1* ^mut^ AML

**FIGURE 4:**
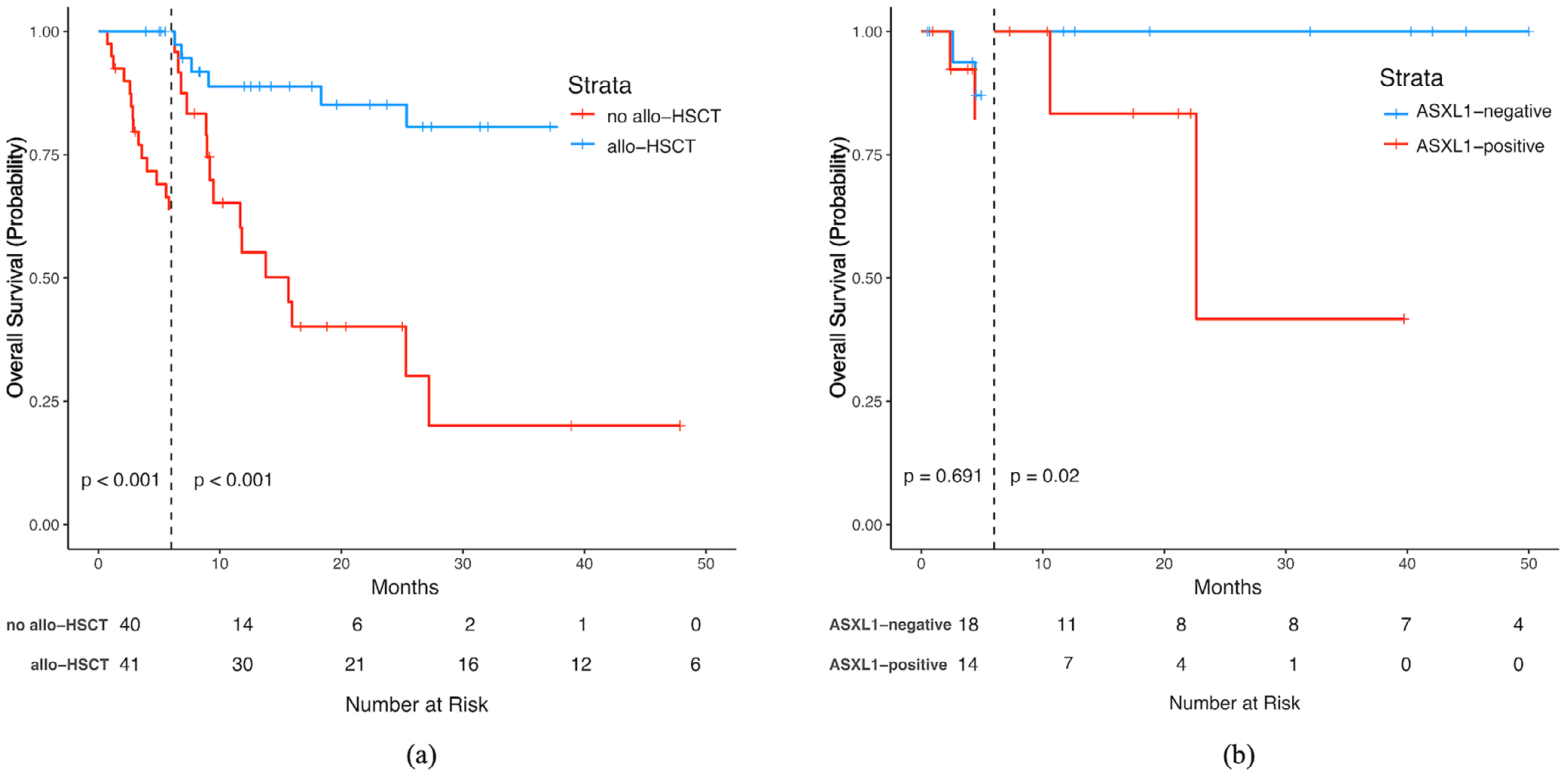
(a) Landmark analysis of OS stratified by allo‐HSCT status in the entire cohort, with the landmark time set at 6 months post‐diagnosis. (b) Post‐transplant OS analysis in the allo‐HSCT subgroup, stratified by pre‐transplant *ASXL1* status, with landmarking at 6 months post‐HSCT. OS, overall survival; allo‐HSCT, allogeneic hematopoietic stem cell transplantation. VAF, variant allele frequency.

Forty‐one patients received allo‐HSCT and 21 (21/41, 51.2%) underwent the procedure within the first 6 months after diagnosis. The median OS of patients receiving allo‐HSCT was not reached, whereas that of patients not receiving allo‐HSCT was only 8.9 months. To address time‐related bias of allo‐HSCT, a landmark analysis was conducted with a landmark time of 6 month post diagnosis. The analysis revealed a statistically significant difference in OS between patients who underwent allo‐HSCT and those who did not, both before and after the 6‐month landmark time (*p* < 0.001; Figure 4) Considered as a time‐dependent covariate, allo‐HSCT predicted superior OS (HR = 0.234, 95% CI 0.088–0.626, *p* = 0.004) in patients with *ASXL1* mutations and those with a 2022 ELN adverse risk (HR = 0.266, 95% CI 0.097–0.729, *p* = 0.010). Our findings suggest that allo‐HSCT could improve survival in AML patients with mutated *ASXL1*, which is in accordance with the findings of a previous study on *ASXL1* ^mut^ AML [16]. Among these 41 patients, 20 patients received induction therapy of HMA–venetoclax, 14 of IC, and seven of HMA + IAG/HAG. However, posttransplant OS did not differ significantly across initial treatment groups (HMA + VEN vs. IC vs. HMA + HAG/IAG; *p* = 0.903, Figure S2), which indicated induction regimen did not independently influence post‐HSCT survival in ASXL1‐mutated AML. In addition, allo‐HSCT was performed in 17 patients who did not achieve CR/CRi in the first cycle. Eleven of them were in CR/CRi pretransplant and six cases underwent salvage transplants without prior remission. For patients who did not achieve CR/CRi in the first cycle, those who proceeded to allo‐HSCT had significantly prolonged survival compared to those who did not (median OS, not reached vs. 5.6 months; HR = 0.206, 95% CI 0.061–0.693, *p* = 0.011). Moreover, *ASXL1* mutation influenced the prognosis of patients receiving allo‐HSCT, as we found patients with positive *ASXL1* mutations detected before transplant had significantly shorter post‐transplant survival after a six‐month landmark time than those with undetectable ASXL1 (*p* = 0.02).

## 4 Discussion

Intensive chemotherapy has remained the standard care for patients with AML, the benefit of which is greatly limited to patients without adverse‐risk cytogenetics and mutations. New regimens are needed for patients with adverse‐risk factors, including *ASXL1* mutations. Studies on the effect of HMAs together with venetoclax on patients with *ASXL1* ^mut^ AML are rare. To the best of our knowledge, this was the first study to compare the impact of HMAs combined with venetoclax on the response and clinical outcomes of *ASXL1* ^mut^ AML patients ≤ 60 years old with that of patients with IC, albeit retrospectively. We also reported the associations between the G646fs variant and the efficacy of HMA–venetoclax induction and the role of allo‐HSCT in *ASXL1* ^mut^ AML.

In this study with a pool of 81 patients, compared with IC, the induction of HMA–venetoclax independently predicted a better response and OS. When 15 patients with favorable cytogenetics or mutations were excluded, the HMA–venetoclax combination still improved the treatment response and prognosis of patients with adverse ELN risk. Our findings suggest that HMAs combined with venetoclax have advantages over traditional IC in younger patients with *ASXL1* ^mut^ AML, especially in the ELN adverse‐risk subgroup. Research is needed to clarify the underlying mechanism, and one study revealed that mutated *ASXL1* enhances sensitivity to venetoclax and azacytidine via epigenetic upregulation of bcl‐2 and DNA methylation alterations [17]. Notably, HMAs combined with venetoclax as consolidation were not able to predict OS or RFS compared with the use of a cytarabine‐based regimen. Given the retrospective nature of our study, prospective studies are needed to validate the efficiency of HMAs combined with venetoclax in AML patients with *ASXL1* mutations. In addition, according to 2024 ELN recommendations [8], *IDH1/IDH2* mutations are associated with favorable prognosis and *TP53* with unfavorable outcome in AML patients treated non‐intensively. Molecular profiling of 31 patients receiving HMA + venetoclax induction revealed complete absence of *TP53* mutations and 23.3% IDH1/2 incidence (7/31). IDH1/2 status remained non‐predictive for CR (OR = 2.118, 95% CI 0.210–21.389, *p* = 0.525) or OS (HR = 0.907, 95% CI 0.094–8.785, *p* = 0.933), which may be due to the limited sample size. Future studies with larger cohorts are warranted to validate these observations.

Admittedly, HMA–venetoclax is not standard therapy for younger AML patients without comorbidities. However, emerging evidence supports the efficacy of HMA–venetoclax in younger cohorts. Chen et al. conducted a phase 2 clinical trial on decitabine combined with venetoclax as an induction treatment for young fit patients with AML, where decitabine and venetoclax showed superior efficacy and safety to IC [18]. Our study highlights the potential utility of HMA–venetoclax in younger ASXL1‐mutated AML, particularly within the ELN adverse‐risk subgroup. These findings acknowledge the potential role of HMA–venetoclax in adverse‐risk AML regardless of age when IC is predicted to be ineffective. However, it is not emphasized that HMA–venetoclax should supplant IC, and the decision to employ HMA–venetoclax as initial therapy in younger patients with AML warrants careful consideration.

Mutations in *ASXL1* are located before the PHD region and result in a C‐terminal truncated protein [17, 19, 20]. Similar to other reports, we found that the most prevalent mutation was a guanine duplication that gave rise to a frameshift (p. Gly646TrpfsX12) in *ASXL1* [21, 22, 23]. In this study, the G646fs variant was not able to predict a worse prognosis for all 81 patients, which was in accordance with previous studies [14, 16]. However, in 26 patients receiving HMA–venetoclax, G646fs was associated with a lower CR/CRi rate and worse EFS, which indicated that the G646fs variant may have a different pathogenetic mechanism than other variants. The correlation between the efficiency of venetoclax and the G646fs variant needs to be further investigated.

Currently, allo‐HSCT is the only cure for AML. Zhou et al. [16] found that allo‐HSCT effectively improved the prognosis of patients with *ASXL1*‐mutated AML, which was in accordance with our finding. We also noticed that patients with *ASXL1* positivity detected before the transplantation were associated with worse OS after allo‐HSCT than those having *ASXL1* mutation turning negative. Although Heuser et al. suggested that posttransplantation mutations on hematopoiesis–associated genes *DNMT3A*, *TET2*, and *ASXL1* had no prognostic impact in patients with AML [24], dynamic evaluation of pre‐ and post‐transplant *ASXL1* should be made to fully explore the prognostic effect of *ASXL1* burden on allo‐HSCT.

In conclusion, this retrospective study revealed that HMAs and venetoclax induction effectively improved the response and survival of patients with *ASXL1* mutations, and shed light on venetoclax treatment for younger patients and for those categorized into the ELN adverse‐risk subgroup. Clinical trials are in demand, and more personalized treatments are expected with respect to AML patients with mutations in *ASXL1*.

## Author Contributions

**Yiming Cai:** formal analysis, investigation, methodology, writing – original draft, visualization, writing – review and editing. **Jingwen Rui:** writing – original draft, writing – review and editing, methodology, investigation, formal analysis, data curation. **Zhengwen Ding:** formal analysis, writing – original draft, writing – review and editing, investigation, methodology, data curation. **Judan Xie:** data curation, writing – review and editing, formal analysis. **Zhou Jin:** investigation, data curation, writing – review and editing, formal analysis. **Jinyan Xiao:** conceptualization, data curation, writing – review and editing, methodology, supervision, project administration. **Yang Xu:** conceptualization, data curation, funding acquisition, writing – review and editing, methodology, supervision, project administration.

## Ethics Statement

The study protocol was performed in accordance with the Declaration of Helsinki. This study was approved and an exemption was granted by the Ethics Committee of the First Affiliated Hospital of Soochow University. Approval number: 2024685.

## Conflicts of Interest

The authors declare no conflicts of interest.

## Supporting information


**Figure S1.** (a) Genetic landscape of 81 AML patients with *ASXL1* mutation. (b) Number of comutations in *ASXL1* ^mut^ AML categorized by gene function. (c) Most frequently comutated genes in *ASXL1* ^mut^ AML. *ASXL1* ^mut^, *ASXL1* mutation; AML, acute myeloid leukemia.
**Figure S2.** Survival curves of posttransplant OS grouped by the induction therapy. OS, overall survival; HMA, hypomethylating agents; VEN, venetoclax; IC, intensive chemotherapy; IAG, idarubicin, cytarabine, and granulocyte colony‐stimulating factor; HAG, homoharringtonine, cytarabine, and granulocyte colony‐stimulating factor.

## Data Availability

Data in the study are available from the corresponding author upon reasonable request.
