# Correction to “Better Response and Prognosis of Venetoclax Plus Hypomethylating Agents Over Intensive Chemotherapy in Young Adults With Newly Diagnosed ASXL1‐Mutated Acute Myeloid Leukemia”

**DOI:** 10.1002/cam4.71141

**Published:** 2025-08-08

Cai Y, Rui J, Ding Z, Xie J, Jin Z., Xiao J., Xu Y. Better Response and Prognosis of Venetoclax Plus Hypomethylating Agents Over Intensive Chemotherapy In Young Adults With Newly Diagnosed ASXL1‐Mutated Acute Myeloid Leukemia. *Cancer Med*. 2025 Jul;14(14): e71037.

In the published version of this article, the first authorship was listed solely as Yiming Cai. The first authorship attribution was incomplete. The authorship statement should be added: “Yiming Cai, Jingwen Rui, and Zhengwen Ding contributed equally to this work.”

We apologize for this error.

