# Supplementary material for: Better Response and Prognosis of Venetoclax Plus Hypomethylating Agents Over Intensive Chemotherapy in Young Adults With Newly Diagnosed ASXL1 ‐Mutated Acute Myeloid Leukemia
Source: Cancer Med. 2025 Jul 18;14(14):e71037. doi: 10.1002/cam4.71037 (PMC12272307; doi:10.1002/cam4.71037)
Supplement: Supplementary file 1 — Figure S1. (a) Genetic landscape of 81 AML patients with ASXL1 mutation. (b) Number of comutations in ASXL1 mut AML categorized by gene function. (c) Most frequently comutated genes in ASXL1 mut AML. ASXL1 mut, ASXL1 mutation; AML, acute myeloid leukemia. Figure S2. Survival curves of posttransplant OS grouped by the induction therapy. OS, overall survival; HMA, hypomethylating agents; VEN, venetoclax; IC, intensive chemotherapy; IAG, idarubicin, cytarabine, and granulocyte colony‐stimulating factor; HAG, homoharringtonine, cytarabine, and granulocyte colony‐stimulating factor. [file CAM4-14-e71037-s001.docx]

**Supplementary Material**

**Better response and prognosis of venetoclax plus hypomethylating agents over intensive chemotherapy in young adults with newly diagnosed *ASXL1*-mutated acute myeloid leukemia**

Yiming Cai^#1,2^, Jingwen Rui^#1,2^, Zhengwen Ding^#1,2^, Judan Xie^1,2^, Zhou Jin^1,2^, Jinyan Xiao^*1,2^, Yang Xu^*1,2^

1. Jiangsu Institute of Hematology, National Clinical Research Center for Hematologic Diseases, The First Affiliated Hospital of Soochow University, Suzhou, P. R. China.
2. Institute of Blood and Marrow Transplantation, Collaborative Innovation Center of Hematology, Soochow University, Suzhou, P. R. China.

*Corresponding Author:

Jinyan Xiao, M.S.

Address: Jiangsu Institute of Hematology, Key Laboratory of Thrombosis and Hemostasis of the Ministry of Health, The First Affiliated Hospital of Soochow University, No.188 Shizi Street, Suzhou 215006, Jiangsu Province, P. R. China.

E-mail: xiao_jin_yan@163.com

Yang Xu, M.D.

Address: Jiangsu Institute of Hematology, Key Laboratory of Thrombosis and Hemostasis of the Ministry of Health, The First Affiliated Hospital of Soochow University, No.188 Shizi Street, Suzhou 215006, Jiangsu Province, P. R. China.

E-mail: xuyang1020@126.com


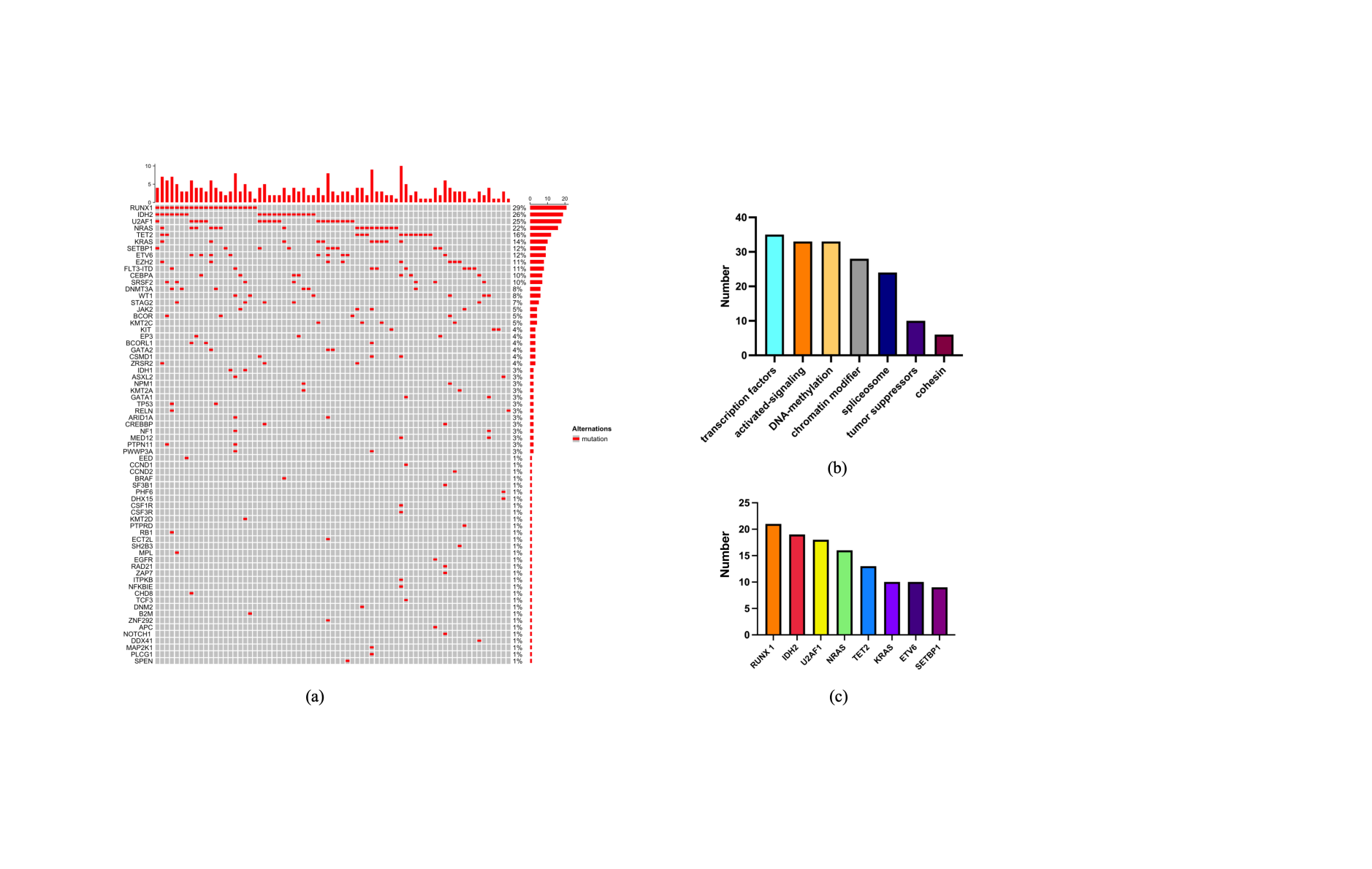


**Figure S1.** (a) Genetic landscape of 81 AML patients with *ASXL1* mutation. (b) Number of co-mutations in *ASXL1*^mut^ AML categorized by gene function. (c) Most frequently co-mutated genes in *ASXL1*^mut^ AML. *ASXL1*^mut^, *ASXL1* mutation; AML, acute myeloid leukemia.

**
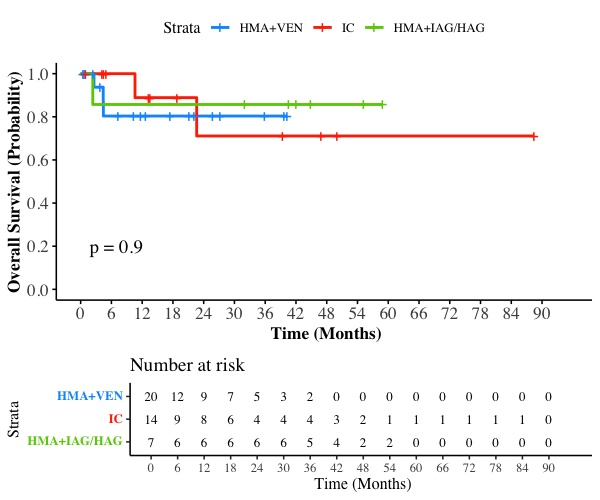
**

**Figure S2.** Survival curves of post-transplant OS grouped by the induction therapy. OS, overall survival; HMA, hypomethylating agents; VEN, venetoclax; IC, intensive chemotherapy; IAG, idarubicin, cytarabine and granulocyte colony stimulating factor; HAG, homoharringtonine, cytarabine and granulocyte colony stimulating factor.
